# Rolling Bearing Fault Diagnosis Based on Multi-Source Domain Joint Structure Preservation Transfer with Autoencoder

**DOI:** 10.3390/s26010222

**Published:** 2025-12-29

**Authors:** Qinglei Jiang, Tielin Shi, Xiuqun Hou, Biqi Miao, Zhaoguang Zhang, Yukun Jin, Zhiwen Wang, Hongdi Zhou

**Affiliations:** 1 China Nuclear Power Operation Technology Corporation, Ltd., Wuhan 430223, China; jiangql@cnnp.com (Q.J.); houxq@cnnp.com (X.H.); miaobq@cnnp.com (B.M.); zhangzg04@cnnp.com (Z.Z.); 2 State Key Laboratory of Digital Manufacturing Equipment and Technology, Huazhong University of Science and Technology, Wuhan 430074, China; tlshi@hust.edu.cn; 3 School of Mechanical Engineering, Hubei University of Technology, Wuhan 430068, China; 102400015@hbut.edu.cn (Y.J.); wzw2797391196@163.com (Z.W.)

**Keywords:** multi-source domain, joint structure preservation, similarity measure, autoencoder, distribution alignment, fault diagnosis

## Abstract

Domain adaptation methods have been extensively studied for rolling bearing fault diagnosis under various conditions. However, some existing methods only consider the one-way embedding of original space into a low-dimensional subspace without backward validation, which leads to inaccurate embeddings of data and poor diagnostic performance. In this paper, a rolling bearing fault diagnosis method based on multi-source domain joint structure preservation transfer with autoencoder (MJSPTA) is proposed. Firstly, similar source domains are screened by inter-domain metrics; then, the high-dimensional data of both the source and target domains are projected into a shared subspace with different projection matrices, respectively, during the encoding stage. Finally, the decoding stage reconstructs the low-dimensional data back to the original high-dimensional space to minimize the reconstruction accuracy. In the shared subspace, the difference between source and target domains is reduced through distribution matching and sample weighting. Meanwhile, graph embedding theory is introduced to maximally preserve the local manifold structure of the samples during domain adaptation. Next, label propagation is used to obtain the predicted labels, and a voting mechanism ultimately determines the fault type. The effectiveness and robustness of the method are verified through a series of diagnostic tests.

## 1. Introduction

As one of the core components of rotating machinery systems, the rolling bearing’s operational state is directly related to the safe operation of the transmission system. Fault diagnosis in rolling bearings has great practical significance [1]. Various fault diagnosis methods for rolling bearings have been proposed. However, the working state of the bearing is complex, with inconsistent fault signal distribution and imbalanced fault categories [2].

Transfer learning (TL) reduces the cost of data acquisition and annotation by transferring the knowledge acquired from the source domain to the target domain [3]. TL methods are extensively applied in the field of fault diagnosis [4]. Ding et al. [5] proposed a deeply imbalanced domain-adaptive framework to address feature shift and label shift under varying operating conditions. Li et al. [6] designed a deep self-supervised data processing network for unknown scenarios, using neighborhood clustering and feature alignment methods to separate domain-known and domain-unknown samples. Qian et al. [7] presented a relationship transfer domain generalization network to reduce distribution discrepancies between source and unknown target domains, thereby addressing the domain confusion problem. Xing et al. [8] proposed a diagnostic method for multi-classifier integrated adaptive knowledge transfer. Yang et al. [9] designed a cross-domain diagnostic model with multi-layer domain adaptation and pseudo-label learning.

The above deep TL methods are of great interest and have high diagnostic accuracy for both single-source domain transfer tasks and target domain diagnostic tasks containing a small number of labeled samples. However, single-source transfer requires high similarity between the two domains, which may lead to ‘negative transfer’ if the distribution difference is large. Multi-source data (e.g., different working conditions and equipment types) can be integrated with fault characteristics information from diverse sources to enhance diagnostic accuracy [10]. In addition, deep TL methods require substantial labeled data for training, which makes the tuning process time consuming and costly.

Furthermore, most existing data-driven methods treat vibration signals merely as numerical vectors, neglecting the underlying physical mechanism of rolling bearings. Faults typically manifest as transient impulses in the time domain or energy shifts in specific frequency bands [11,12]. Therefore, effective transfer learning should not only align statistical distributions but also preserve the manifold structure of these physical characteristics. The proposed MJSPTA aims to bridge this gap by integrating graph embedding with autoencoders to preserve the intrinsic manifold structure of these physical fault characteristics during the transfer process, ensuring that the impulsive fault signatures are not lost during domain adaptation.

Feature-based approaches [13,14] aim to mitigate the distribution differences between source and target domains by extracting shared features between source and target domains to achieve better generalization capabilities. Meanwhile, the sample-based approach [15] selects source domain samples relevant to the target domain, thereby enhancing domain adaptation. Evidence [16,17] indicates that a combined feature- and sample-based transfer learning approach can achieve effective knowledge transfer and model-adaptive enhancement with little reliance on any a priori assumptions in the presence of significant distributional differences between the source and target domains. Wu et al. [18] proposed a multi-source domain adaptation network model that dynamically adjusts parameters based on input sample distributions. Chen et al. [19] designed a multi-source weighted diagnostic model that uses a weighted learning strategy to adaptively assign weights for feature distribution alignment between known-class and unknown-class samples. Fan et al. [20] presented a hybrid domain generalization model using a difference metric to measure intra- and inter-domain distances. Chen et al. proposed an adversarial domain-invariant generalization framework to obtain domain-invariant features via adversarial learning [21]. Wang et al. [22] structured a multi-source domain feature-adaptive framework for time-varying operating conditions, which uses both intra-domain and inter-domain alignment strategies to reduce the distributional differences between domains.

Although the aforementioned methods have achieved promising results in transfer learning-based fault diagnosis, two critical limitations remain: (1) Rigid Feature Alignment: Most methods employ a single shared projection matrix for both the source and target domains. However, when the distribution discrepancy is significant (e.g., due to large speed variations), a shared projection lacks the flexibility to capture domain-specific characteristics, which constrains the model’s generalization ability and impedes the extraction of truly domain-invariant features. (2) Lack of Structure Validation: During dimensionality reduction, existing approaches typically rely on a unidirectional mapping from the high-dimensional space to the latent subspace. This one-way process lacks a backward validation mechanism. Consequently, the intrinsic manifold structure of the original data may be distorted or lost in the low-dimensional space, leading to inaccurate embeddings that fail to represent the fault features faithfully.

Herein, a rolling bearing fault diagnosis method called multi-source domain joint structure preservation transfer with autoencoder (MJSPTA) is proposed in this paper. In the MJSPTA framework, a bidirectional mapping is constructed by leveraging the autoencoder paradigm to validate the accuracy of low-dimensional embeddings. Within the shared subspace, the marginal and conditional distributions of the two aligned domains are matched via the Maximum Mean Discrepancy (MMD) metric. Meanwhile, sample neighborhood relationships are preserved through distributional alignment and sample weighting, and the local manifold structure of the data is retained by integrating graph embedding theory with Fisher’s criterion. In summary, the main contributions of this work can be outlined as follows:

(1) MJSPTA is a domain adaptation method that enables joint migration of feature and sample knowledge. Specifically, it mitigates the distribution discrepancy between domains in terms of feature dimensions, and the neighborhood relationship of samples is maintained from the sample-specific perspective.

(2) MJSPTA employs two distinct projection matrices: one tailored to the source domain and the other specific to the target domain. The autoencoder-integrated model proposed herein exhibits enhanced performance in cross-domain tasks.

(3) The autoencoder framework integrated into MJSPTA enhances the accuracy of subspace mapping, thereby yielding low-dimensional embeddings that more faithfully characterize the original data.

The paper is structured as follows: Section 2 describes the related work of MJSPTA. Section 3 introduces a rolling bearing fault diagnosis method based on MJSPTA. Section 4 provides experimental comparisons between the proposed method and existing methods, along with parametric analyses. Conclusions are summarized in Section 5.

## 2. Preliminaries

### 2.1. Problem Definition

Given labeled source domains $\left\{X_{s},Y_{s}\right\}$ and one unlabeled target domain $X_{n}$, where $X_{s}\epsilon R^{d_{s}\times n_{s}}$ and $X_{n}\epsilon R^{d_{n}\times n_{n}}$ are the source and target domain samples, $n_{s}$ and $n_{n}$ are the total number of samples in the source and target domains, $d_{s}$ and $d_{n}$ are their dimensions, $Y_{s}$ is the label of the source domain, and $P\left(X_{s}\right)\neq P\left(X_{n}\right)$, $P\left(Y_{s}|\;X_{s}\right)\neq$$P\left(Y_{n}|\;X_{n}\right).$

### 2.2. Similarity Between Domains

When performing multi-source domain selection, the Maximum Mean Discrepancy (MMD) [23] is used to quantify the similarity between source and target domains; a larger MMD value indicates lower similarity between domains. The inter-domain similarity between source and target domains can be defined as:

(1) $$MMD(X_{s},X_{n})={\left\|\frac{1}{n_{s}}\sum_{i=1}^{n_{s}}\varphi (X_{s}^{p})-\frac{1}{n_{n}}\sum_{j=1}^{n_{n}}\varphi (X_{n}^{q})\right\|}_{H}^{2}=tr(UK)$$

where *H* denotes the Reproducing Kernel Hilbert Space (RKHS), φ denotes the nonlinear mapping of the kernel space, $\varphi (X_{s}^{p})$ and $\varphi (X_{n}^{q})$ denote the transformed features, and K is the Gram matrix of the source and target domains, where U is the MMD matrix defined as:

(2) $$U_{ij}=\left\{\begin{array}{cc} \frac{1}{n_{s}^{2}}, & \mathrm{if}\;x_{i},x_{j}\in X_{s}\\ \frac{1}{n_{n}^{2}}, & \mathrm{if}\;x_{i},x_{j}\in X_{n}\\ -\frac{1}{n_{s}n_{n}}, & \mathrm{otherwise}\;(\mathrm{one}\;\mathrm{sample}\;\mathrm{from}\;X_{s},\;\mathrm{one}\;\mathrm{from}\;X_{n}) \end{array}\right.$$

### 2.3. Graph Embedding and Fisher’s Discriminant Criterion

Graph embedding [24] inherently preserves local sample consistency during dimensionality reduction.

(3) $$min\frac{1}{2}\sum_{i,j}W_{ij}{\left\|x_{i}-x_{j}\right\|}_{2}^{2}=\frac{1}{2}\left(\sum_{i}x_{i}^{T}x_{i}\sum_{j}W_{ij}-2\sum_{i,j}W_{ij}x_{i}^{T}x_{i}\right)=minTr\left(XLX^{T}\right)$$

Here, $X=\left[x_{1},\;x_{2},\;\ldots ,x_{N}\right],\;x_{i}\in R^{D}$ is the training set, *N* is the number of samples, and *D* is the dimension of training samples. For Laplacian Eigenmaps [25], firstly, an adjacency graph G that connects nodes i and j is constructed if $x_{i}$ and $x_{j}$ are “close”, usually using the k-Nearest Neighbor method, in order to construct graph G. Secondly, the weights $W_{ij}$ of any two neighbors $x_{i}$ and $x_{j}$ in graph G are set. O=D−W, where D is the diagonal matrix and the diagonal elements $D_{ii}=\sum_{j\neq i}W_{ij}$.

Linear Discriminant Analysis (LDA), which is based on Fisher’s discriminant criterion, is a classical supervised dimensionality reduction method. It is aimed at finding an optimal projection direction $W_{opt}=\left[w_{1},w_{2},\ldots ,w_{d}\right]$ that maximizes the separation between different bearing state classes while it minimizes the scatter within each class in the projected space:

(4) $$W_{opt}=\arg\underset{W}{\max}\;\frac{\left|W^{T}S_{b}W\right|}{\left|W^{T}S_{w}W\right|}$$

Here, *d* is the subspace dimension, where *c* denotes the class label, and $n_{c}$ is the number of samples in the *c*-th class, where $S_{b}=\sum_{c=1}^{C}n_{c}\left(u_{c}-u\right)\left(u_{c}-u\right)$ and $S_{w}=\sum_{c=1}^{C}\sum_{j=1}^{n_{c}}\left(x_{j}^{c}-u_{c}\right)\left(x_{j}^{c}-u_{c}\right)$ are the between-class and within-class scatter matrices, respectively. Here, *C* is the number of classes, $n_{c}$ is the number of samples belonging to the c-th class, the $x_{j}^{c}$ denotes the *j*-th sample in the *c*-th class, and the $u_{c}$ and u are the mean vectors of feature vectors for class *c* and all samples, respectively.

### 2.4. Autoencoder

An autoencoder is a classical neural network designed to precisely reconstruct input data, which is aimed at distilling critical fault features from high-dimensional bearing vibration signals while discarding redundant information. Autoencoders comprise an input layer, a hidden layer, and an output layer.

For bearing fault diagnosis, autoencoders learn compressed representations of vibration signals through an encoder–decoder architecture to identify subtle fault patterns based on distinctive reconstruction behavior. Normal vibration signals exhibit low reconstruction errors due to stable operational patterns, whereas fault-induced signals generate significantly higher errors caused by anomalous transient impulses. This characteristic enables the reconstruction error itself to serve as a direct quantifiable fault indicator. The central optimization objective is formalized as:

(5) $$\underset{W,W^{\prime }}{\min}\sum_{i=1}^{N}{\left\|x_{i}-{\tilde{x}}_{i}\right\|}^{2}$$

where $R\left(x_{i},{\tilde{x}}_{i}\right)={\left\|x_{i}-{\tilde{x}}_{i}\right\|}^{2}$ denotes the reconstruction loss for training sample *i*, which is optimized by adjusting encoder weights W and decoder weights $W^{\prime }$ and where ${\tilde{x}}_{i}$ denotes the reconstructed output of the input sample $x_{i}$.

## 3. Framework for MJSPTA

**Figure 1:**
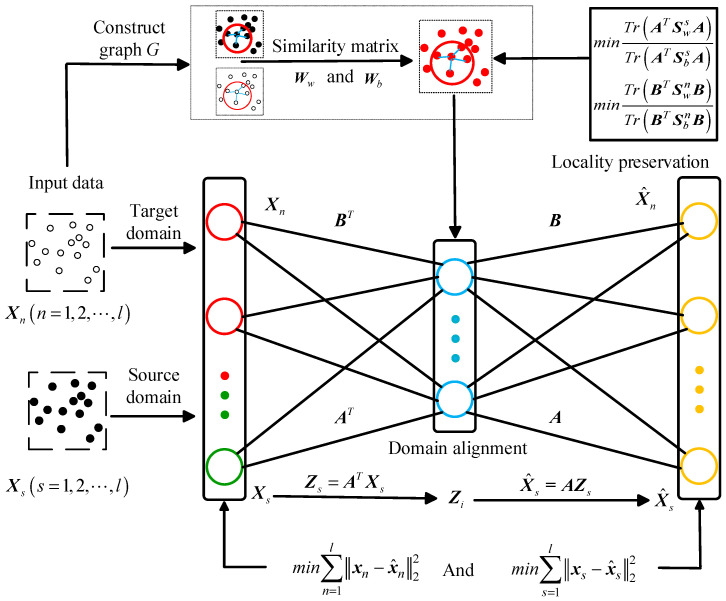
Bidirectional mapping framework.

The concept of autoencoders has been extensively applied in the field of neural networks since the 1980s and has driven the development of deep learning in its early stages [26,27]. With the advent of emerging techniques, including dropout [28] and batch normalization [29], deep neural networks have eliminated the necessity for autoencoder-based greedy layer-wise pre-training [30] in modern learning paradigms. However, the ability of autoencoders to perform dimensionality reduction and unsupervised feature learning retains its utility. A standard autoencoder comprises a two-layer fully connected neural network, including an input layer, a hidden layer, and an output layer. The encoder consists of the input layer and an encoding (hidden) layer, whereas the decoder comprises a decoding (hidden) layer and the output layer. The encoder transforms input data into latent representations, while the decoder reconstructs the original inputs from these feature embeddings. The optimal feature representation of the input data is obtained by minimizing the reconstruction error and training the autoencoder by adjusting its weight parameters. In an autoencoder, if the number of nodes in the hidden layer is less than that in the input layer, it is termed an under-complete model; if it exceeds that of the input layer, it is called an over-complete model. Moreover, if the activation function of the hidden layer is linear, the model is called a linear autoencoder. MJSPTA employs an under-complete nonlinear autoencoder with a single hidden layer. The bidirectional autoencoder mapping framework proposed in this paper is illustrated in Figure 1.

### 3.1. Distributed Alignment

Most existing methods [31,32] rely on a single shared projection for both domains, which fails to fully eliminate the inter-domain bias. To construct a domain-invariant subspace and learn shared latent features, MJSPTA computes two distinct projection matrices: A for the source domain and B for the target domain. Data from both domains are projected into their corresponding subspaces through matrices A and B, which effectively aligns the two domains. In this context, the low-dimensional embedding can be viewed as the autoencoder’s coding component. The source domain data is projected into the d-dimensional subspace through $A\epsilon R^{d_{s}\times d}$, where $d=\min\left(d_{s},d_{n}\right)$. $Z_{s}=A^{T}X_{s}$ is denoted as the low-dimensional data projected from the source domain, while the unlabeled target domain data is denoted low-dimensionally as $Z_{n}=B^{T}X_{n}$. The reconstruction process, which validates the low-dimensional embeddings by mapping them back to the original space, is given by ${\hat{X}}_{s}=AZ_{s}$ and ${\hat{X}}_{n}=BZ_{n}$.

The marginal and conditional distributions between the source and target domains are aligned via the MMD:

(6) $$\begin{array}{c} \begin{array}{c} min\\ A,B \end{array} \end{array}\sum_{s=1}^{l}\sum_{n=1}^{l}{\left\|X-\hat{X}\right\|}_{2}^{2}+D_{MD}\left(X_{s},X_{n},A,B\right)+D_{CD}\left(X_{s},X_{n},A,B\right)$$

where $X_{n}$ is an unlabeled sample of the target domain. $D_{MD}$ and $D_{CD}$ are the marginal and the conditional cross-domain data distribution, respectively. For simplicity, $D_{MD}$ and $D_{CD}$ are set to the same weights, and the marginal distribution discrepancy term $D_{MD}$ can be expressed as:

(7) $$D_{MD}={\left\|\frac{1}{n_{s}}\sum_{i=1}^{n_{s}}A^{T}x_{s}^{i}-\frac{1}{n_{n}}\sum_{j=1}^{n_{n}}B^{T}x_{n}^{j}\right\|}^{2}$$

The conditional distribution discrepancy term $D_{CD}$ is expressed as:

(8) $$\begin{array}{c} D_{CD}=\overset{C}{\underset{c=1}{\sum }}{\left\|\frac{1}{n_{s}^{c}}\overset{n_{s}^{c}}{\underset{i=1}{\sum }}A^{T}x_{s}^{i,c}-\frac{1}{n_{n}^{c}}\overset{n_{n}^{c}}{\underset{j=1}{\sum }}B^{T}x_{n}^{j,c}\right\|}^{2}+\overset{C}{\underset{c=1}{\sum }}\left(\frac{1}{n_{s}^{c}n_{n}^{c}}\overset{n_{s}^{c}}{\underset{i=1}{\sum }}\overset{n_{n}^{c}}{\underset{j=1}{\sum }}{\left\|A^{T}x_{s}^{i,c}-B^{T}x_{n}^{j,c}\right\|}^{2}\right) \end{array}$$

$n_{n}$ is the number of unlabeled target domain samples, and *C* denotes the total number of classes. $n_{s}^{c}$ and $n_{n}^{c}$ denote the number of source and target domain samples belonging to the c-th class, respectively. In $D_{CD}$, the first term calculates the difference in class means. The second term preserves neighborhood relationships to maintain proximity among same-class samples. As target domain labels are unavailable, label propagation [33,34] provides initial pseudo-labels, which are updated based on model predictions during optimization.

For simplicity, the source domain reconstruction error is expressed as:

(9) $$\begin{array}{l} J_{MSE}^{S}=\sum_{s=1}^{l}{\left\|X-\hat{X}\right\|}_{2}^{2}=\sum_{s=1}^{l}{\left\|x_{s}-AA^{T}x_{s}\right\|}_{2}^{2}=\sum_{s=1}^{l}{\left\|\left(I-AA^{T}\right)x_{s}\right\|}_{2}^{2}\\ =Tr\left(\left(I-AA^{T}\right){x_{s}x_{s}}^{T}{\left(I-AA^{T}\right)}^{T}\right)=Tr\left(E\right) \end{array}$$

Accordingly, the target domain reconstruction error can be expressed here as:

(10) $$J_{MSE}^{N}=Tr\left(\left(I-BB^{T}\right){x_{n}x_{n}}^{T}{\left(I-BB^{T}\right)}^{T}\right)=Tr\left(F\right)$$

Here, $E=\left(I-AA^{T}\right){x_{s}x_{s}}^{T}{\left(I-AA^{T}\right)}^{T}$, and the target domain $F=\left(I-BB^{T}\right){x_{n}x_{n}}^{T}{\left(I-BB^{T}\right)}^{T}$.

### 3.2. Sample Reweighting

The domain adaptation objective minimizes distributional discrepancies between source and target domains by assigning weights parameterized by vectors α and β, respectively, leading to the objective function:

(11) $$\begin{array}{c} min\\ A,B \end{array}D_{MD}\left(\alpha ,\beta ,X_{s},X_{n},A,B\right)+D_{CD}\left(\alpha ,\beta ,X_{s},X_{n},A,B\right)+\alpha Tr\left(E\right)+\beta Tr\left(F\right)$$

$$s.t.\left\{\alpha_{i}^{c},\beta_{i}^{c}\right\}\in \left[0,1\right],\frac{\alpha^{c^{T}}1_{n_{s}^{c}}}{n_{s}^{c}}=\frac{\beta^{c^{T}}1_{n_{n}^{c}}}{n_{n}^{c}}=\sigma$$

where $\alpha =\left[\alpha^{1};\ldots \alpha^{c};\ldots \alpha^{C}\right]\in R^{n_{s}}$ and $\beta =\left[\beta^{1};\ldots \beta^{c};\ldots \beta^{C}\right]\in R^{n_{n}}$ are the weights of the samples in the source and target domains, respectively. $1_{n_{s}^{c}}$ and $1_{n_{n}^{c}}$ are column vectors with all ones. σ is the proportion of landmark samples in the entire source domain as well as the target domain. $D_{MD}$ and $D_{CD}$ can be further expressed as:

(12) $$\begin{array}{c} D_{MD}=A^{T}X_{s}C_{sm}X_{s}^{T}A+B^{T}X_{n}C_{nm}X_{n}^{T}B-2A^{T}X_{s}C_{snm}X_{n}^{T}B \end{array}$$

(13) $$D_{CD}=A^{T}X_{s}C_{sc}X_{s}^{T}A+B^{T}X_{n}C_{nc}X_{n}^{T}B-2A^{T}X_{s}C_{snc}X_{n}^{T}B$$

where C is the coefficient matrix, $C_{sm}=\frac{1}{\delta^{2}n_{s}^{2}}\alpha •\alpha^{T}$, $C_{nm}=\frac{1}{\delta^{2}n_{n}^{2}}\beta •\beta^{T}$, $C_{snm}=\frac{1}{\delta^{2}n_{s}n_{n}}\alpha •\beta^{T}$, and $C_{sc}$, $C_{nc}$, and $C_{snc}$ are diagonal matrices. The objective function can be converted to:

(14) $$\begin{array}{l} \begin{array}{c} min\left(A^{T}M_{ss}A+B^{T}M_{nn}B-2A^{T}M_{sn}B+\alpha Tr\left(E\right)+\beta Tr\left(F\right)\right) \end{array}\\ =\begin{array}{c} min\\ A,B \end{array}Tr\left(\left[A^{T}B^{T}\right]\left[\begin{array}{cc} M_{ss} & M_{sn}\\ M_{ns} & M_{nn} \end{array}\right]\left[\begin{array}{c} A\\ B \end{array}\right]\right)+\alpha Tr\left(E\right)+\beta Tr\left(F\right) \end{array}$$

where $M_{ss}=X_{s}\left(C_{sm}+C_{sc}\right)X_{s}^{T}$, $M_{nn}=X_{n}\left(C_{nm}+C_{nc}\right)X_{n}^{T}$, $M_{sn}=X_{s}\left(C_{snm}+C_{snc}\right)X_{n}^{T}$.

### 3.3. Local Manifold Structure Preservation

To jointly retain the intrinsic manifold structure and enhance class discriminability within the data, our method integrates a Laplacian graph regularization term and a Fisher discriminant term. This combined formulation significantly improves the model’s cross-domain generalization ability, as defined by:

(15) $$min\frac{Tr\left(A^{T}X_{s}L_{w}^{s}X_{s}^{T}A\right)}{Tr\left(A^{T}X_{s}L_{b}^{s}X_{s}^{T}A\right)}=min\frac{Tr\left(A^{T}S_{w}^{s}A\right)}{Tr\left(A^{T}S_{b}^{s}A\right)}$$

(16) $$min\frac{Tr\left(B^{T}X_{n}L_{w}^{n}X_{n}^{T}B\right)}{Tr\left(B^{T}X_{n}L_{b}^{n}X_{n}^{T}B\right)}=min\frac{Tr\left(B^{T}S_{w}^{n}B\right)}{Tr\left(B^{T}S_{b}^{n}B\right)}$$

where $S_{b}^{s}=X_{s}L_{b}^{s}X_{s}^{T}$, $S_{w}^{s}=X_{s}L_{w}^{s}X_{s}^{T}$, $S_{b}^{n}=X_{n}L_{b}^{n}X_{n}^{T}$, and $S_{w}^{n}=X_{n}L_{w}^{n}X_{n}^{T}$, and where $L_{w}^{s}$, $L_{w}^{n}$, $L_{b}^{s}$, and $L_{b}^{n}$ are the Laplace matrices of the source and target domains and the intrinsic and penalty maps, respectively. $W_{w}$ and $W_{b}$ are the weight matrices of the intrinsic and penalty maps, respectively, which can effectively maintain the local manifold structure of the global discriminative information of the data. They are constructed as follows:

(1) $W_{w}$: For sample $x_{i}$, if sample $x_{j}$ has the same label as $x_{i}$, the nearest neighbor pairs of $x_{i}$ and $x_{j}$ are connected.

(2) $W_{b}$: For each domain, k-nearest vertex pairs are connected, and the samples in each vertex pair belong to a different class.

By applying (1), samples from the same class can be made more compact and retain the local manifold structure. By applying (2), samples from the same domain but different classes can be made more separable, and global discriminative information can be preserved. In this paper, $W_{w}$ and $W_{b}$ are calculated with Gaussian kernel functions:

(17) $$W_{ij}=\left\{\begin{array}{ll} e^{\left(-\left({\left\|x_{i}-x_{j}\right\|}^{2}\right)/2\right)}, & x_{i}\neq x_{j}\\ 0 & x_{i}=x_{j} \end{array}\right.$$

### 3.4. Objective Function

In summary, the objective function is:

(18) $$\begin{array}{c} min\\ A,B \end{array}\frac{Tr\left(\left[A^{T}\;\;B^{T}\right]\left[\begin{array}{cc} M_{ss}+\gamma S_{w}^{s} & M_{sn}\\ M_{ns} & M_{nn}+\gamma S_{W}^{n}+\mu I \end{array}\right]\left[\begin{array}{c} A\\ B \end{array}\right]\right)}{Tr\left(\left[A^{T}\;\;B^{T}\right]\left[\begin{array}{cc} \gamma S_{b}^{s} & 0\\ 0 & \gamma S_{b}^{n}+\mu S_{h}^{n} \end{array}\right]\left[\begin{array}{c} A\\ B \end{array}\right]\right)}$$

where

(19) $$S_{h}^{n}=X_{n}\left(I_{n}-\frac{1}{n_{n}}1_{n_{n}}1_{n_{n}}^{T}\right)X_{n}^{T}$$

is the covariance matrix in the target domain; γ and μ are the balance parameters of the local holdout and target variance terms, respectively. To solve the objective function to $P=\left[A;B\right]$, the objective function can be rewritten as:

(20) $$\underset{P}{\max}Tr\left(\left[P^{T}\right]\left[\begin{array}{cc} \gamma S_{b}^{s} & 0\\ 0 & \gamma S_{b}^{n}+\mu S_{h}^{n} \end{array}\right]\left[P\right]\right)$$

$$s.t.Tr\left(\left[P^{T}\right]\left[\begin{array}{cc} M_{ss}+\gamma S_{w}^{s} & M_{sn}\\ M_{ns} & M_{nn}+\gamma S_{w}^{n}+\mu I \end{array}\right]\left[P\right]\right)=1$$

According to constrained optimization theory, the Lagrange multiplier method [35] is used to introduce the Lagrange multiplier Φ. The Lagrange function of Equation (20) is Equation (21), where $\Phi =\mathrm{diag}\left(\phi_{1},\cdots ,\phi_{d}\right)$ is the diagonal matrix of the d largest eigenvalues corresponding to the eigen-decomposition of Equation (22).

(21) $$\Gamma =Tr\left(P^{T}\left[\begin{array}{cc} S_{b}^{s} & 0\\ 0 & \gamma S_{b}^{n}+\mu S_{h}^{n} \end{array}\right]P\right)-Tr\left(\left(P^{T}\left[\begin{array}{cc} M_{ss}+\gamma S_{w}^{s} & M_{sn}\\ M_{ns} & M_{nn}+\gamma S_{w}^{n}+\mu I \end{array}\right]P-I\right)\Phi \right)$$

Therefore, P consists of the d eigenvectors corresponding to Equation (22), and by solving the generalized eigenvalue problem in Equation (22), one obtains the subspace into which A and B are mapped.

(22) $$\left[\begin{array}{cc} \gamma S_{b}^{s} & 0\\ 0 & \gamma S_{b}^{n}+\mu S_{h}^{n} \end{array}\right]P=\left[\begin{array}{cc} M_{ss}+\gamma S_{w}^{s} & M_{sn}\\ M_{ns} & M_{nn}+\gamma S_{W}^{n}+\mu I \end{array}\right]$$

In summary, first, the original data matrix X and similarity matrix W are constructed; then, the autoencoder is trained using the Adam optimization algorithm, setting the learning rate η=0.001. By applying a segmented linear learning rate scheduling strategy with a descent factor of 0.1, the loss function change threshold is ε=0.005, and the momentum coefficient is ρ=0.9. The method proposed herein can be summarized in the following steps:

Input: Source and target domain data: $X_{s}$, $X_{n}$, $Y_{s}$; the similarity matrix $W=\left(W_{ij}\right)$.

Initialize: Parameter *δ=0.5 * (preset based on Gaussian kernel characteristics and verified by preliminary experiments); parameters d (subspace dimension), μ (target variance factor), and *γ* (local retention factor) are determined through a grid search combined with sensitivity analysis, with search ranges d∈[1,10], $\mu \in \left[10^{-3},1\right]$, and $\gamma \in \left[10^{-5},10^{-2}\right]$ (see Section 4.3 Parametric Analysis); set the gradient descent learning rate to η=0.001 and the loss function change threshold to ε=0.005 (referring to classical autoencoder training settings and adjusted by model convergence performance).

**Step 1**: Perform label propagation using $X_{s}$, $X_{n}$, and $Y_{s}$ to initialize the pseudo-labels of the target domain unlabeled data $\;{\hat{y}}_{n}$; compute $S_{h}^{n}$, $M_{nn}$, $M_{sn}$, $M_{ns}$, $S_{b}^{s}$ $S_{w}^{s}$, $S_{b}^{n}$, and $S_{w}^{n}$ according to Equations (14)–(16) and (19), respectively.
**while not converge do**
  **Step 2**: To solve the eigen-decomposition problem in Equation (22), select the *d* eigenvectors corresponding to the *d* largest eigenvalues as Transformation P to obtain Transformations A and B.
  **Step 3**: Map the raw data to the corresponding subspace to obtain an embedded representation of the data: $\begin{array}{c} Z_{s}=A^{T}X_{s} \end{array}$ and $Z_{n}=B^{T}X_{n}$.
  **Step 4**: Perform label propagation on $Z_{s}$, $Z_{n}$, and $Y_{s}$ to update the pseudo-labels of the target domain.
**Step 5**: Update α and β by Equations (11) and (12); update $M_{ss}$, $M_{nn}$, $M_{sn}$, $M_{ns}$, $S_{b}^{n}$, and $S_{w}^{n}$ by Equations (14)–(16); update μ and γ by Equations (18).
**Step 6**: Calculate the objective function Γ using Equation (21) until the reconstruction loss computed by Equations (9) and (10) is <ε.

Output: Predictive labels ${\hat{y}}_{n}$ for unlabeled data in the target domain.

### 3.5. MJSPTA-Based Application

**Figure 2:**
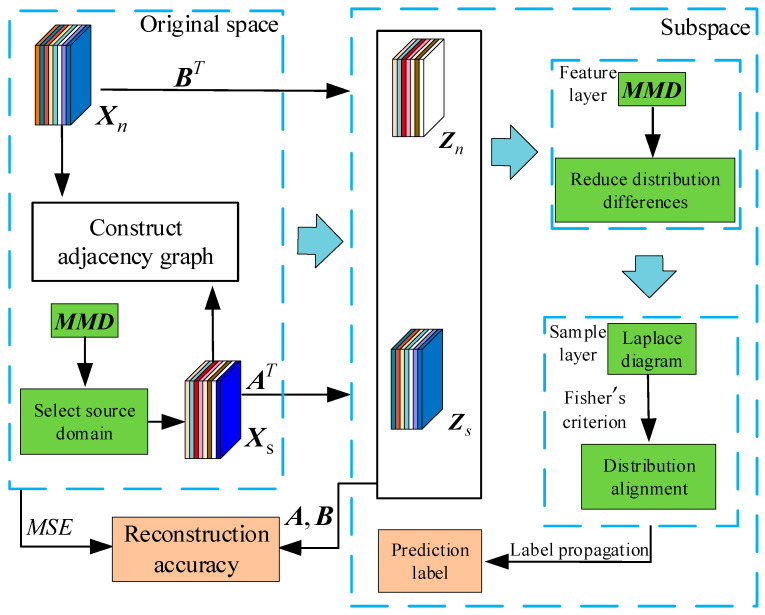
Overall framework of the proposed method.

The diagnostic flow based on the MJSPTA method proposed in this paper is shown in Figure 2, which consists of three main phases: distribution alignment, sample weighting, and local structure preservation. For source domain selection, the MMD metric is employed to identify source domains similar to the target domain and filter out those with significant distributional differences. For the input high-dimensional source and target domain data, we first construct the adjacency graph G; then, the Laplacian Eigenmaps are introduced as the encoding part, which maintains the relationship between the samples by mapping the two-domain data into subspaces through matrices A and B. The decoder validation results in more accurate low-dimensional embeddings. The algorithm model is constructed by Equation (6). At the feature layer, the marginal and conditional distributions between the two domains are aligned using MMD. In the sample layer, two graph Laplacian terms preserve the local manifold structure of the samples, and Fisher’s discriminant criterion achieves the effect of intra-class compactness and inter-class separation. Meanwhile, label propagation is performed utilizing the labeled source domain data to predict labels for the unlabeled target domain samples. Finally, the voting mechanism is used to determine the type of faults in order to enhance result reliability.

### 3.6. Complex Analysis

While MJSPTA demonstrates superior diagnostic performance, its computational complexity warrants discussion. The complexity is primarily dominated by two steps: the graph construction ($O(N^{2}d)$) and the generalized eigen-decomposition ($O(N^{3})$), where N is the sample size and d is the feature dimension. Despite this, the MMD-based source domain selection effectively reduces the input scale N by filtering out irrelevant domains. In our experiments, the average inference time is approximately 25 ms per sample, which satisfies real-time monitoring requirements. A primary limitation, however, is the quadratic complexity $O(N^{2})$ of the Laplacian graph, which may become a bottleneck for extremely large-scale datasets. In future work, we plan to explore approximation techniques such as Nyström sampling or integrate Deep Graph Neural Networks (GNNs) to improve scalability.

## 4. Experimental Verification

### 4.1. Dataset Description

(1) Experiment (1) (Cross-domain bearing fault diagnosis on the same device)

**Figure 3:**
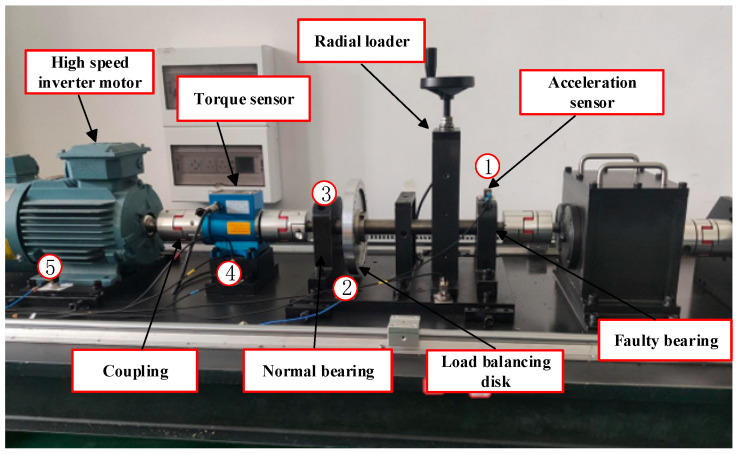
Rolling bearing vibration test rig.

The validity of the proposed method is verified using the test rig shown in Figure 3. This rig mainly includes the motor, coupling, bearing housing, gearbox, and brake. The test bearing is NSK6205; the operating speed includes 1800 r/min and 2400 r/min; the radial load includes 0 N, ±600 N, and the sampling frequency is 10,240 Hz. Inner and outer ring cracks are artificially introduced by using wire cutting, at depths of 0.2 mm and 0.5 mm. Therefore, this experiment primarily involves five distinct working conditions, with each condition comprising 100 samples; each sample has a length of 1024. Six different working conditions (A1, A2…A6) are set up according to different loads and rotational speeds, and their detailed information is shown in Table 1. A multi-source domain transfer experiment task is established to validate the model performance (see Table 2 and Table 3).

(2) Experiment (2) (Cross-equipment bearing fault diagnosis)

Experiment (2) utilizes bearing datasets from different devices, including data from Case Western Reserve University (CWRU) [36], the American Society for Mechanical Failure Prevention Technology (MFPT) [37], Jiangnan University (JNU) [38], and our own test data. The details are shown in Table 4. The configurations of datasets are summarized in Table 4, and the results of source domain selection for Experiment (2) are presented in Table 5. For the CWRU dataset, the test bearing is an SKF6205 deep groove ball bearing. The collected vibration signals form the drive-end bearing; the IN and OU fault diameters are 0.36 mm, and the sampling frequency is 12 kHz. In the MFPT dataset, the fault size is 0.38 mm, the speed is 1500 r/min, and the sampling frequencies for Normal, OU, and IN conditions are 97,656 Hz, 97,656 Hz, and 48,828 Hz, respectively. The Normal and OU radial loads for dataset F are 270 lbs, and the IN loads are 250 lbs and 300 lbs, respectively. In the JNU dataset, the experimental speeds were 600 r/min and 800 r/min, and the sampling frequencies were 50 kHz. N205 bearings were used for normal, outer ring faults and rolling faults. NU205 bearings with detachable outer rings were used for the inner ring faults, with inner and outer ring fault dimensions measuring 0.3 × 0.25 mm (width × depth). The self-test dataset is derived from vibration signals collected from the faulty bearing, with a fault depth of 0.2 mm and a sampling frequency of 10,240 Hz. Three types of faults are selected for each dataset: the normal, the inner ring fault (IN), and the outer ring fault (OU), totaling 300 samples. The experimental results of cross-equipment diagnosis are displayed in Table 6.

### 4.2. Experimental Results

To validate the effectiveness of the proposed method, it is compared with several traditional machine learning methods and transfer learning (TL) methods, including k-Nearest Neighbors (KNNs) [39], Support Vector Machines (SVMs) [40], Domain Adversarial Neural Networks (DANNs) [41], Geodesic Flow Kernel (GFK) [42], Transfer Component Analysis (TCA) [43], and Semi-Supervised Transfer Component Analysis (SSTCA) [44].

(1) Implementation Details:

As suggested in the literature [45], the traversal optimization method is employed to grid search for the optimal parameter settings. For MJSPTA, the fixed average weight parameter σ=0.5, $k\left(x_{i},x_{j}\right)=\exp\left(-\left({\left\|x_{i}-x\right\|}^{2}\right)/2\right)$ is utilized as the kernel function. The kernel function of SVM is $k\left(x_{i},x_{j}\right)=\exp\left(-{\left\|x_{i}-x_{j}\right\|}^{2}/2\sigma^{2}\right)$ with a penalty factor C = 1; the optimal number of nearest neighbors of the KNN method is selected in $\left[1,5,9,13,17,21,25,29,33,63\right]$. In TCA, the optimal hyper-parameters are through Bayesian optimization, with the parameter μ searched in the range of $\left[10^{-3},10^{3}\right]$, and the range of the dimension of the subspace in the range of $\left[1,10\right]$. For SSTCA, the parameter $\gamma_{1}$ is in the range of $\left[10^{-3},1\right]$, and the parameter $\lambda_{1}$ is in the range of $\left[10^{-3},10^{3}\right]$. Moreover, TCA and SSTCA both utilize a linear kernel function and SVM for construction of the model, with C set to 1. The parameter setting of GFK is based on the literature [42], employing nearest neighbors as the classifier; the parameter setting of TLPP is based on the literature [46], optimizing parameters k, l, and λ within the range of $\left[10^{-3},10^{3}\right]$ by using the linear kernel function and SVM classifiers. For DANN, the training batch size is set to 64, and the learning rate is 0.001, as recommended in the literature [47]. The detailed hyper-parameter settings are summarized in Table 7.

(2) Experimental Results:

In Experiment (1), the results of multi-source domain selection are presented in Table 2. The three source domains with the greatest similarity to the target domain were selected for the transfer task experiment, and the diagnostic results are shown in Table 3. Most of the transfer learning (TL) algorithms in the comparison set performed similarly to the traditional machine learning methods, achieving accuracies mostly in the range of 80–90%. However, the proposed MJSPTA method significantly outperformed them, achieving an accuracy of 98.63%. The reason for this superior performance is that MJSPTA enhances the separability of the samples by constructing graph embeddings and applying Fisher’s discriminant criterion. This approach reduces intra-class distances and increases inter-class distances while retaining the local sample consistency, making prediction results more reliable. Additionally, MJSPTA incorporates a voting mechanism to further improve reliability.

In Experiment (2), the results of source domain selection are presented in Table 5, and the results of cross-domain diagnosis are displayed in Table 6. Even though the source and target domain data originated from different devices, the average recognition rate of the proposed MJSPTA method still achieved 98.93%, surpassing all the compared methods. This superior performance is attributed to MJSPTA’s more accurate low-dimensional mapping, which considers bidirectional mapping. MJSPTA utilizes distinct projection matrices for the source and target domains and Fisher’s criterion to identify shared fault features across domains. This approach preserves the local manifold structure and discriminative information of the data while minimizing the domain discrepancies in the subspace.

The superior performance of MJSPTA is closely related to its ability to align with the vibrational dynamics and physical fault characteristics of bearings.

For early weak faults (e.g., inner ring fault with 0.2 mm depth), MJSPTA’s local manifold structure preservation module retains the transient impulse features of vibration signals, which are the core physical signatures of early faults. This ensures that even subtle fault characteristics are not lost during domain adaptation, leading to near-perfect classification accuracy (e.g., 100.00% for the A2 condition in Experiment (1). In contrast, comparison methods such as TCA and DANN neglect these physical features, resulting in lower accuracy for early faults.

The MMD-based distribution alignment aligns the energy distribution of vibration signals in characteristic frequency bands between source and target domains. This ensures that the physical essence of fault signals is consistent across domains, especially in cross-equipment experiments where bearing models and operating conditions differ.

The low standard deviation of MJSPTA (±0.10% in Experiment (1); ±0.16% in Experiment (2) reflects its stability in capturing consistent physical fault characteristics, while comparison methods with higher standard deviations (e.g., DANN ±0.71% in Experiment (1) are more susceptible to non-physical noise interference, indicating weaker robustness in extracting physical fault features.

**Figure 4:**
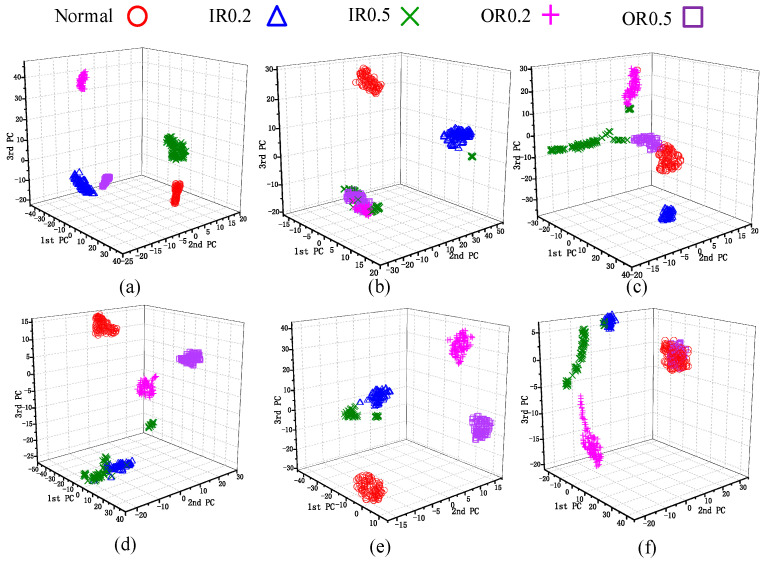
A6 → A1 (**a**) MJSPTA. (**b**) SSTCA. (**c**) DANN. (**d**) KNN. (**e**) GFK. (**f**) TLPP.

**Figure 5:**
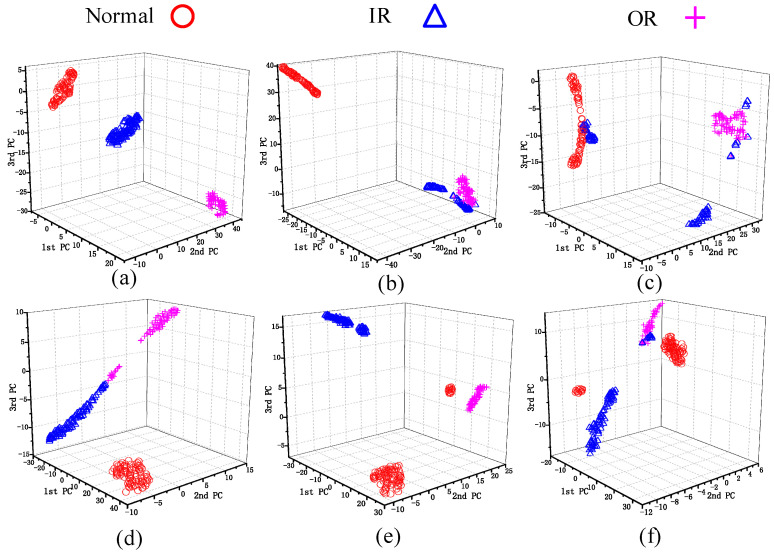
Z1 → A1 (**a**) MJSPTA. (**b**) SSTCA. (**c**) DANN. (**d**) KNN. (**e**) GFK. (**f**) TLPP.

To visualize the data processing effects, two representative tasks were selected: the multi-source domain transfer task A6 → A1 and the cross-equipment transfer task Z1 → A1. The transformed fault features were projected into a 3D space using the t-Distributed Stochastic Neighborhood Embedding (t-SNE) [48] algorithm and visualized as scatter plots, as shown in Figure 4 and Figure 5, respectively. As shown in Figure 4, after domain adaptation via multiple transfer learning methods, most fault categories are separated more distinctly. However, similar faults exhibit poor clustering. There is overlap between normal and outer ring 0.5 mm samples, making it difficult to distinguish the faults. From Figure 5, it is apparent that the clustering performance of the compared methods is inferior to MJSPTA, with significantly poorer class separability. The specific reasons are as follows: the method proposed in this paper reduces distributional differences between the data of the two domains, preserves the local manifold structure of the samples, and explores the domain-shared features by jointly aligning the source and target domains at both the feature level and the sample level. This approach makes the fault features more distinguishable and representative.

To quantitatively evaluate the clustering performance shown in Figure 4 and Figure 5, we calculated the Trace of Between-Class Scatter ($Tr(S_{b})$) and Trace of Within-Class Scatter ($Tr(S_{w})$). As shown in Table 8, the Fisher ratio $J=Tr(S_{b})/Tr(S_{w})$ for MJSPTA is 434.69, which is substantially higher than TCA (196.13) and DANN (75.36). This quantitative result confirms that MJSPTA yields the most compact intra-class structure and separable inter-class margins.

### 4.3. Parametric Analysis

To further analyze the impact of the target variance factor μ and the local retention factor γ as well as the subspace dimension d on the fault diagnosis results, parametric analyses of μ and γ were conducted. According to the multi-source domain selection method, the optimal set of source domains was obtained and three transfer tasks Z1 → A1, Z2 → A3 and X4 → A5 were randomly selected for parameter analysis.

**Figure 6:**
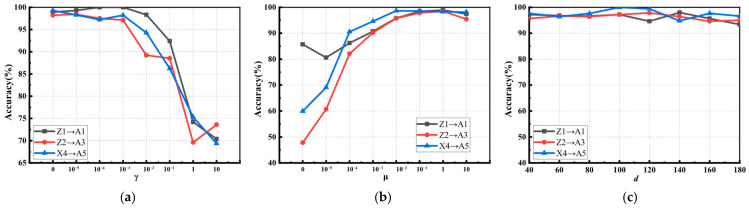
Parameter sensitivity: (**a**) results with varying γ; (**b**) results with varying μ; and (**c**) results with varying d.

The effect of the variation of the local preservation factor γ on the recognition accuracy is shown in Figure 6a; the value of γ should be set smaller in the interval $\left[10^{-5},10^{-2}\right]$.

The target variance term aims to optimize the feature mapping by maintaining the variance properties of the target domain, helping the model to maintain the distribution and internal structure of the data in the target domain. By optimizing the target variance term, the model can effectively adapt to the data distribution in the target domain and can enhance its generalization ability. The influence of parameter μ is shown in Figure 6b, and the recognition accuracy increases monotonically as μ is increasing. Therefore, in the illustration, the optimal range of μ is $\;\left[10^{-3},1\right]$.

As shown in Figure 6c, MJSPTA is not sensitive to the value of the subspace embedding dimension d. The reason is that the target variance term stabilizes the feature mapping at the global distribution level; the local holdout term provides fault tolerance at the local structure level, constrains the manifold structure in the feature space, and preserves the similarity relationships of neighboring samples. This joint parameter optimization makes the model more flexible and practical for selecting subspace dimensions in engineering.

## 5. Conclusions

To address the challenges posed by the substantial differences in feature distribution between rolling bearing domains and the scarcity of labels in target domains under variable operating conditions, we introduce a novel fault diagnosis method called multi-source domain joint structure preservation transfer with autoencoder (MJSPTA). The proposed method leverages multi-source domain joint structure-preserving transfer and is augmented with an autoencoder.

(1) Firstly, an inter-domain similarity metric is employed to identify relevant source domains. This approach mitigates the challenges of negative transfer and high computational costs incurred by uniformly selecting features from all source domains in multi-source domain transfer diagnosis.

(2) In the graph embedding-based dimensionality reduction, an autoencoder is utilized to map the low-dimensional embeddings to the high-dimensional space, which is crucial for validating the dimensionality reduction performance through the evaluation of reconstruction accuracy.

(3) Furthermore, to tackle the structural and distributional variability across domains, graph embedding and Fisher’s criterion are employed. These techniques mitigate cross-domain discrepancies, extract domain-shared structural features, and preserve the integrity of the manifold structure of the data.

Despite the superior performance of MJSPTA in rolling bearing fault diagnosis, the adaptability to extreme conditions and heterogeneous data needs further exploration. Therefore, future research will focus on integrating physical prior knowledge of bearing vibration dynamics (e.g., transient impulse characteristics) into the model to enhance the diagnosis of early weak faults. Moreover, an adaptive parameter optimization framework will be developed to realize dynamic adjustment of key parameters such as μ and γ based on data distribution.

## Figures and Tables

**Table 1:** Dataset of rolling bearings in different operating conditions on the same equipment.

Datasets	Fault Type/Size	Speed (rpm)/Load (N)	Sample Size
A1	Normal, inter 0.2, inter 0.5, outer 0.2, outer 0.5	1800/0	500
A2		1800/600	500
A3		1800/−600	500
A4		2400/0	500
A5		2400/600	500
A6		2400/−600	500

**Table 2:** Selection of similar source domains based on MMD distance for Experiment (1) (Note: Lower values indicate higher similarity; “-” indicates that the distance is not calculated for the same domain).

	A1	A2	A3	A4	A5	A6
A1	-	7.17× 10^−12^	2.55 × 10^−13^	2.58 × 10^−13^	2.55 × 10^−13^	2.55 × 10^−13^
A2	7.17 × 10^−12^	-	7.21 × 10^−12^	7.17 × 10^−12^	7.17 × 10^−12^	7.17 × 10^−12^
A3	2.55 × 10^−13^	7.21 × 10^−12^	-	2.15 × 10^−16^	2.15 × 10^−16^	2.15 × 10^−16^
A4	2.58 × 10^−13^	7.17 × 10^−12^	2.15 × 10^−16^	-	7.51 × 10^−20^	1.61 × 10^−21^
A5	2.55 × 10^−13^	7.17 × 10^−12^	2.15 × 10^−16^	7.51 × 10^−20^	-	7.50 × 10^−20^
A6	2.55 × 10^−13^	7.17 × 10^−12^	2.15 × 10^−16^	1.61 × 10^−21^	7.50 × 10^−20^	-
Results	A3\A4\A6	A1\A4\A5	A4\A5\A6	A3\A5\A6	A3\A4\A6	A3\A4\A5

**Table 3:** Experimental results of the same equipment (Accuracy: Mean% ± Std).

	SVM	KNN	TCA	GFK	SSTCA	TLPP	DANN	MJSPTA
A1	80.00 ± 0.42	88.40 ± 0.35	78.40 ± 0.38	90.60 ± 0.25	80.00 ± 0.41	94.69 ± 0.22	80.58 ± 0.75	99.80 ± 0.11
A2	76.60 ± 0.45	97.20 ± 0.15	82.00 ± 0.32	89.00 ± 0.28	91.80 ± 0.25	89.56 ± 0.26	90.80 ± 0.62	100.00 ± 0.00
A3	60.00 ± 0.58	89.20 ± 0.31	78.00 ± 0.40	93.00 ± 0.21	57.00 ± 0.55	92.64 ± 0.24	80.23 ± 0.81	99.80 ± 0.12
A4	95.40 ± 0.25	74.00 ± 0.48	89.40 ± 0.29	85.41 ± 0.33	93.20 ± 0.28	91.82 ± 0.25	85.61 ± 0.68	100.00 ± 0.00
A5	76.80 ± 0.46	71.60 ± 0.52	95.60 ± 0.18	80.00 ± 0.35	92.40 ± 0.29	89.94 ± 0.28	75.42 ± 0.72	94.60 ± 0.22
A6	61.60 ± 0.55	92.32 ± 0.24	86.90 ± 0.33	93.20 ± 0.22	94.80 ± 0.26	95.36 ± 0.19	77.23 ± 0.70	97.60 ± 0.14
Avg.	75.07 ± 0.45	85.45 ± 0.34	85.05 ± 0.32	88.54 ± 0.27	84.87 ± 0.34	92.34 ± 0.24	81.65 ± 0.71	98.63 ± 0.10

**Table 4:** Dataset of rolling bearing operating conditions for different equipment.

Data Sources	Datasets	Fault Type	Speed (rpm)/Load	Sample Size
CWRU	X1	Normal/IN/OU	1797/0 HP	300
	X2		1772/1 HP	300
	X3		1750/2 HP	300
	X4		1730/3 HP	300
MFPT	Y1	Normal/IN/OU	1500/270 lbs (IN250lbs)	300
	Y2		1500/270 lbs (IN300lbs)	300
JNU	Z1	Normal/IN/OU	600	300
	Z2		800	300

**Table 5:** Selection of similar source domains based on MMD distance (Note: Lower values indicate higher similarity).

	A1	A2	A3	A4	A5	A6
X1	0.0449	0.0449	0.0449	0.0449	0.0449	0.0449
X2	0.0467	0.0467	0.0467	0.0467	0.0467	0.0467
X3	0.0555	0.0555	0.0555	0.0555	0.0555	0.0555
X4	0.0452	0.0452	0.0452	0.0452	0.0452	0.0452
Y1	3.97 × 10^−24^	1.17 × 10^−23^	8.22 × 10^−27^	2.81 × 10^−33^	8.27 × 10^−35^	8.36 × 10^−36^
Y2	3.97 × 10^−24^	1.17 × 10^−23^	8.22 × 10^−27^	2.81 × 10^−33^	8.27 × 10^−35^	8.36 × 10^−36^
Z1	0.0139	0.0139	0.0139	0.0139	0.0139	0.0139
Z2	1.06 × 10^−5^	1.06 × 10^−5^	1.06 × 10^−5^	1.06 × 10^−5^	1.06 × 10^−5^	1.06 × 10^−5^
Results	Y1\Y2\Z2	Y1\Y2\Z2	Y1\Y2\Z2	Y1\Y2\Z21	Y1\Y2\Z2	Y1\Y2\Z2

**Table 6:** Experimental results of different equipment (Accuracy: Mean % ± Std).

	SVM	KNN	TCA	GFK	SSTCA	TLPP	DANN	MJSPTA
A1	56.36 ± 0.85	66.67 ± 0.62	63.33 ± 0.65	87.46 ± 0.35	74.56 ± 0.55	87.46 ± 0.35	88.76 ± 0.88	99.30 ± 0.18
A2	76.80 ± 0.62	82.63 ± 0.45	65.60 ± 0.58	82.71 ± 0.42	81.70 ± 0.48	92.71 ± 0.28	92.97 ± 0.75	96.40 ± 0.42
A3	66.67 ± 0.75	78.33 ± 0.55	78.73 ± 0.42	79.11 ± 0.45	83.80 ± 0.42	89.11 ± 0.32	61.33 ± 1.10	100.00 ± 0.00
A4	54.60 ± 0.88	80.64 ± 0.48	76.53 ± 0.51	86.87 ± 0.38	80.36 ± 0.46	86.87 ± 0.38	84.77 ± 0.82	100.00 ± 0.00
A5	76.60 ± 0.65	71.60 ± 0.58	75.60 ± 0.53	93.56 ± 0.28	92.40 ± 0.32	93.56 ± 0.25	94.74 ± 0.68	98.40 ± 0.25
A6	68.80 ± 0.72	92.30 ± 0.32	76.90 ± 0.48	90.44 ± 0.33	94.80 ± 0.28	90.44 ± 0.33	93.27 ± 0.74	99.43 ± 0.12
**Average**	66.64 ± 0.75	78.70 ± 0.50	72.78 ± 0.53	86.69 ± 0.37	84.60 ± 0.42	90.03 ± 0.32	85.97 ± 0.83	98.93 ± 0.16

**Table 7:** Hyper-parameter settings and search ranges for comparison methods ("-" indicates not applicable).

Method	Parameter	Symbol	Search Range/Setting	Optimized Value
MJSPTA	Average Weight	σ	Fixed	0.5
	Kernel Function	-	Gaussian	-
SVM	Penalty Factor	C	Fixed	1
	Kernel Bandwidth	σ	Grid Search	(Matched to data)
KNN	Neighbors	k	$\left[1,5,9,\ldots ,63\right]$	9
TCA	Regularization	μ	$\left[10^{-3},10^{3}\right]$	1.0
	Subspace Dim	d	$\left[1,10\right]$	10
SSTCA	Manifold Param	$\gamma_{1}$	$\left[10^{-3},1\right]$	0.01
	Regularization	$\lambda_{1}$	$\left[10^{-3},10^{3}\right]$	0.1
TLPP	Parameters	k,l,λ	$\left[10^{-3},10^{3}\right]$	(Best found)
DANN	Batch Size	-	Fixed	64
	Learning Rate	η	Fixed	0.001

**Table 8:** Between-class scatter and within-class scatter.

	TCA	TLPP	GFK	KNN	SVM	SSTCA	DANN	MJSPTA
$Tr(S_{b})$	176.52	168.27	14417	1245.32	1454.4	145.36	1262.27	1017.17
$Tr(S_{w})$	0.90	2.38	82.78	21.23	18.13	1.18	16.75	2.34
$J=\frac{Tr(S_{b})}{Tr(S_{w})}$	196.13	70.70	174.16	58.66	80.22	123.19	75.36	434.69

## Data Availability

The public datasets (CWRU, MFPT, and JNU) analyzed in this study can be found at the following links: https://engineering.case.edu/bearingdatacenter/download-data-file (accessed on 24 December 2025); https://gitcode.com/Resource-Bundle-Collection/c0691/?utm_source=pan_gitcode&index=top&type=card&uuid_tt_dd=10_4543420140-1755016136567-412320&from_id=143385987&from_link=567003abb851ef52807b4e3fb3b72c49 (accessed on 24 December 2025); and https://github.com/ClarkGableWang/JNU-Bearing-Dataset (accessed on 24 December 2025). Additionally, the data generated from our own bearing test rig experiments are available from the corresponding author upon reasonable request.
